# Effects of voluntary exercise on the expression of browning markers in visceral and subcutaneous fat tissue of normotensive and spontaneously hypertensive rats

**DOI:** 10.1007/s00424-021-02629-9

**Published:** 2021-12-10

**Authors:** Meryem Sevval Karadedeli, Rolf Schreckenberg, Hanna S. Kutsche, Klaus-Dieter Schlüter

**Affiliations:** Physiologisches Institut, Justus-Liebig-University Giessen, Aulweg 129, D-35392, Giessen, Germany

**Keywords:** UCP, Visfatin, Cidea, Nrg4, Hypertension

## Abstract

High physical activity is important to optimize the function of adipose tissue. Dysfunctional adipose tissue contributes to the development of metabolic stress, chronic inflammation, and hypertension. To improve our current understanding of the interaction between physical exercise and adipose tissue, we analyzed the effect of 10 months voluntary running wheel activity of rats on uncoupling protein (UCP) 1 negative white adipose tissue (visceral and subcutaneous adipose tissue, VWAT and SWAT). Analysis was performed via RT-PCR and immunoblot from adipose tissues depicted from adult normotensive and spontaneously hypertensive female rats. UCP1 negative VWAT differed from UCP1 positive WAT and brown adipose tissue (BAT) from interscapular fat depots, by lacking the expression of UCP1 and low expression of Cidea, a transcriptional co-activator of UCP1. High physical activity affected the expression of five genes in SWAT (Visfatin (up), RBP5, adiponectin, Cidea, and Nrg4 (all down)) but only one gene (Visfatin, up) in VWAT. Furthermore, the expression of these genes is differentially regulated in VWAT and SWAT of normotensive and spontaneously hypertensive rats (SHR) under sedentary conditions (UCP2) and exercise (Visfatin, Cidea, Nrg4). Keeping the animals after 6 months of voluntary exercise under observation for an additional period of 4 months without running wheels, Visfatin, Cidea, and Nrg4 were stronger expressed in VWAT of SHRs than in sedentary control rats. In summary, our study shows that SWAT is more responsible to exercise than VWAT.

## Introduction

Obesity is more than ever a raising world health problem. It occurs as the consequence of an imbalance between caloric intake and caloric consumption. Energy is stored predominantly in white adipose tissues (WAT) in the form of triglycerides; however, there are anatomical and functional differences in various types of WAT within the body. In example, subcutaneous WAT (SWAT) and visceral WAT (VWAT) have different localizations and functions. An increase in energy expenditure by exercise can induce a phenotypic change of WAT through a transformation from an energy-storing tissue to a thermogenic beige adipose tissue [1]. This process has been termed browning of WAT. There is evidence that exercise induces browning of SWAT but not VWAT in mice, rats, and humans [1–4]. The release of myokines, that are cytokines or hormones derived from the skeletal muscle, may trigger effects of exercise in adipose tissue [5]. Among the myokines, interleukin (IL)-6 has addressed most attention. Interestingly, IL-6 induces remodeling of adipose tissue. Therefore, release of IL-6 from skeletal muscle may indeed connect exercise to browning of WAT [1].

WAT is also located in close proximity to brown adipose tissue (BAT), like in the interscapular region (IWAT). IWAT located in the vicinity of BAT differs in several aspects from SWAT and VWAT including origin and phenotype. Browning of WAT has initially linked to the expression of uncoupling protein (UCP) 1. UCP1 is a mitochondrial protein which uncouples proton transport from ATP production and directs the chemical energy of the mitochondrial proton gradient into heat (thermogenesis). Browning in its pure sense means an induction of UCP1 expression in WAT located in close proximity of BAT. This type of WAT is then claimed “beige” adipose tissue. Nevertheless, SWAT and VWAT are also affected by exercise. In this context, it must be noted that adaptations of adipose tissue to increased energy demand are not limited to the induction of UCP1 [6]. Furthermore, adipose tissue is not only involved in energy storage and energy supply but also linked to regulation of the metabolic homeostasis, appetite, angiogenesis, immunity, and cardiovascular function. Therefore, a characterization of the effects of exercise on adipose tissue must cover them in a broad way.

Obesity is a risk factor often associated with other risk factors such as hypertension and insulin resistance — together referred to as the metabolic syndrome. Spontaneously hypertensive rats (SHR) have several metabolic differences to normotensive Wistar rats such as reduced expression of CD36, increased norepinephrine turnover in adipose tissue, and insulin insensitivity [7–9]. Although SHRs are lighter in weight compared to normotensive Wistar rats, they show metabolic dysfunction and cardiovascular abnormalities seen also in patients with metabolic syndrome. In this study, we used normotensive Wistar rats to clarify the effect of life-long voluntary exercise on the molecular signature of SWAT and VWAT in rats and compared these results to those generated with SHRs. The study is aimed at improving our current understanding of interaction between physical activity, WAT function, and co-morbidities such as hypertension.

## Material and methods

The investigations are in agreement with the “Guide for the Care and Use of Laboratory Animals” purchased by the US National Institutes of Health (NIH Publication No. 85–23, revised 1996). The study was approved by the local authorities (RP Gießen; V 54–19 c 20 15 h 01 GI 20/1 Nr. 76/2014 and Nr. 77/2014).

### Animal model

Female Wistar rats and SHRs were randomized selected and kept either under standard conventional housing conditions (WIS-Sed and SHR-Sed) or received free access to a running wheel during night starting at the age of 6 weeks (pre-hypertensive state of SHR) and maintained for 10 months before rats were sacrificed (WIS-Run and SHR-Run). In some groups, SHRs access to free running wheels was ceased for the last 4 months (SHR-ExR). Another group of rats had free excess to running wheels for 4 weeks with cessation for another 4 weeks and again access to running wheels (intermittent exercise; SHR-IR). Running distance, running time, kidney function, blood pressure, and adaptation to exercise in kidney and skeletal muscle were reported earlier for these the rats [10–13]. Briefly, running distance was 74 ± 33 km/week (Wistar) and 94 ± 16 km/week (SHR). Running time was 21 ± 9 h/week (Wistar) and 30 ± 4 h/week (SHR). Systolic blood pressure was 127 ± 7 mmHg (Wistar) and 190 ± 11 mmHg (SHR). Diastolic blood pressure was 80 ± 6 mmHg (Wistar) and 122 ± 10 mmHg (SHR) (see Ref. 13 for technical details).

Male or female UCP2^−/−^ rats and their wild-type littermates were used at an age of 3 months to isolate VWAT and SWAT.

### Tissue material

**Fig. 1:**
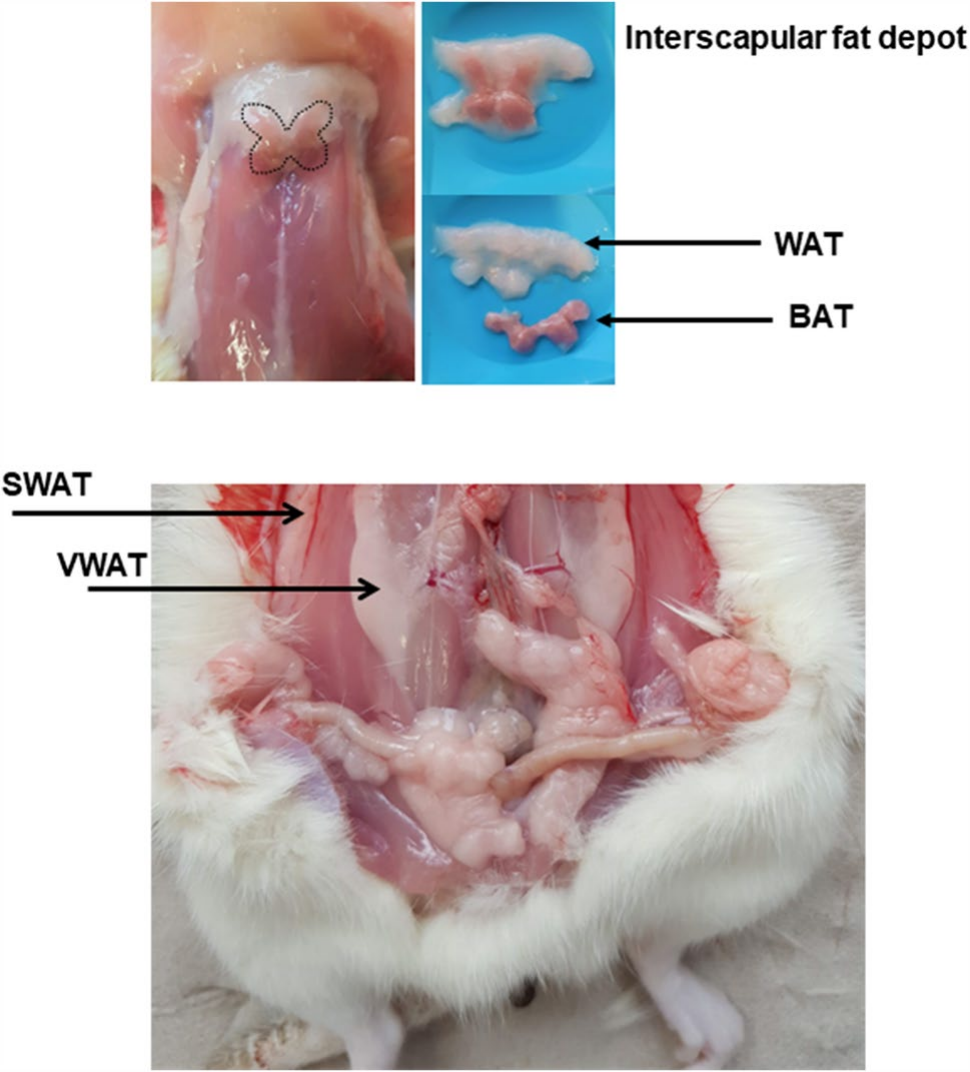
Anatomic localization of the different sources of adipose tissues used in this study (S, subcutaneous; V, visceral; WAT, white adipose tissue; BAT brown adipose tissue)

The study was intended to investigate the effect of free running wheel activity on cardiovascular adaptations. Therefore, rats were anesthetized using isoflurane inhalation and subsequently sacrificed by cervical dislocation at an age of 11.5 months. In addition, we now analyzed molecular adaptation in fat tissue. Therefore, after removing the heart and lung for isolation of the heart, SWAT and VWAT were dissected and quickly frozen in fluid nitrogen and subsequently stored at −80 °C until use (Fig. 1). In the same manner, interscapular BAT and WAT were dissected from fat depots in the neck region and the UCP1 positive WAT was then separated from the BAT.

### RNA isolation and cDNA synthesis

Total RNA was isolated from adipose tissue using peqGold TriFast™ (peqlab, Biotechnologie GmbH, Germany) according to the manufacturer’s protocol. In brief, small pieces of adipose tissue were homogenized in 1 ml TriFAST™ solution for 20 s at 5000 rpm using Precellys® homogenizer. After adding 200 μl chloroform, samples were centrifuged for 10 min at 12500 rpm and the homogenate was allowed to separate into a clear upper aqueous layer (containing RNA), an interphase, and a red lower organic layer. The RNA was precipitated from the aqueous layer with isopropanol at −20 °C. To remove genomic DNA contamination, isolated RNA samples were treated with 1 U DNase/μg RNA (Invitrogen, Karlsruhe, Germany) for 15 min at 37 °C. One microgram of total RNA was used in a 10 μl reaction to synthesize cDNA using Superscript RNaseH Reverse Transcriptase (200 U/μg RNA, Invitrogen, Karlsruhe, Germany) and oligo dTs as primers. RT reactions were performed for 50 min at 37 °C.

### RT-PCR

Quantitative real-time PCR was performed using the CFX Connect Real-Time PCR Detection System (Bio-Rad, Munich, Germany) in combination with IQ SYBR green real-time supermix (Bio-Rad, Munich, Germany) as described before [10]. The sequences of the primers used in this study are indicated in Table 1. Quantification was performed as described before [6], based on the ΔΔC_T_ method.

**Table 1:** Primer sequences used in this study

Gene	Forward	Reverse	Ampl.-length
Adiponectin (NM_144744.3)	GGC CGT TCT CTT CAC CTA CG	TGT CCC CTT CCC CAT ACA CT	122 bp
AR-β3 (NM_013108.2)	GGT TGG GCT ATG CCA ACT CT	CCT GTT GAG CGG TGA GTT CT	176 bp
B2M (NM_012512.2)	GCCGTCGTGCTTGCCATTC	CTGAGGTGGGTGGAACTGAGAC	117 bp
Cidea (NM_001170467.1)	AGA AAT GGA CAC CGG GCA AT	TGA AGC TTG TGC AGC GGA TA	177 bp
Leptin (NM_013076.3)	ACC AGA CCC TGG CAG TCT AT	TTG GAG AAG GCC AGC AGA TG	118 bp
Nrg4 (NM_001191109.1)	CCA GGC ACA GGT CAT TTT GC	AGC TGC CGA CAG GTT ACT TT	161 bp
RBP4 (NM_013162.1)	CAA GGG ACG AGT CCG TCT TC	GTC ATC GTT TCC TCG CTG GA	139 bp
TGF-β1 (NM_021578)	ATT CCT GGC GTT ACC TTG G	CCT GTA TTC CGT CTC CTT GG	117 bp
UCP-1 (NM_012682.2)	ATC TTC TCA GCC GGC GTT TC	AGG GTG GTG ATG GTC CCT AA	146 bp
UCP-2 (NM_019354.2)	CAC CGT CAT TGC CTC CCC CG	CGG AGC ATG GTC AGG GCA CA	102 bp
Visfatin (NM_177928.3)	CTG TGT CTG TGG TCA GCG AT	GTG TCG AGC GGA TTT CCA GA	142 bp

### Western blot

Total protein was extracted from tissues using cell lysis buffer (Cell Signaling, Technology, Frankfurt, Germany), according to the manufacturer’s protocol. Briefly, the homogenates were centrifuged at 14,000 g for 10 min and the supernatants were treated with Laemmli buffer (Sigma-Aldrich, Taufkirchen, Germany). The protein concentration was adjusted to either 4 g/l for tissue extracts or 2 g/l for isolated cells. Protein samples were loaded on NuPAGE Bis-Tris Precast gels (10%; Life Technology, Darmstadt, Germany) and subsequently transferred onto nitrocellulose membranes. The expression of UCP2 was analyzed with an antibody (kindly provided by Prof. Dr. E. Pohl), whose specificity was evaluated before [14, 15]. Expression of UCP2 was normalized to the expression of actin using an antibody produced in rabbit (Cell Signaling, Technology, Frankfurt, Germany). Secondary antibodies (horseradish peroxidase-coupled secondary antibody) directed against rabbit IgG or mouse IgG were purchased from Dako (now Agilent Technologies, Santa Clara, CA, USA).

### Statistics

**Fig. 2:**
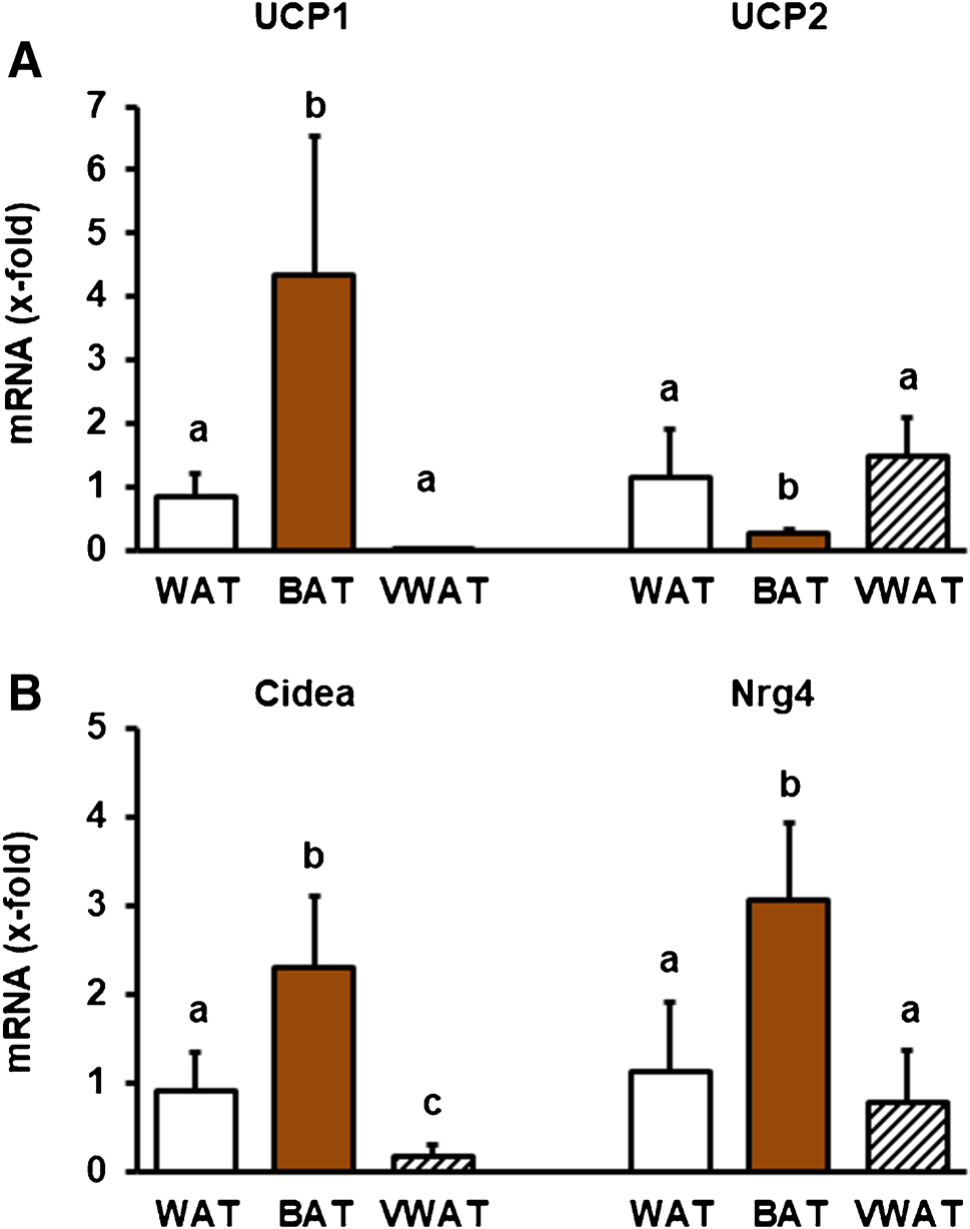
Expression of typical browning markers in WAT and BAT from interscapular fat depots and in visceral white adipose tissue (VWAT). Data are means + S.D.; analysis was performed by ANOVA and Student-Newman-Keuls post hoc analysis. Groups are labeled with identical letters when the difference was >0.05. Different letters indicate group differences with *p* < 0.05

**Fig. 3:**
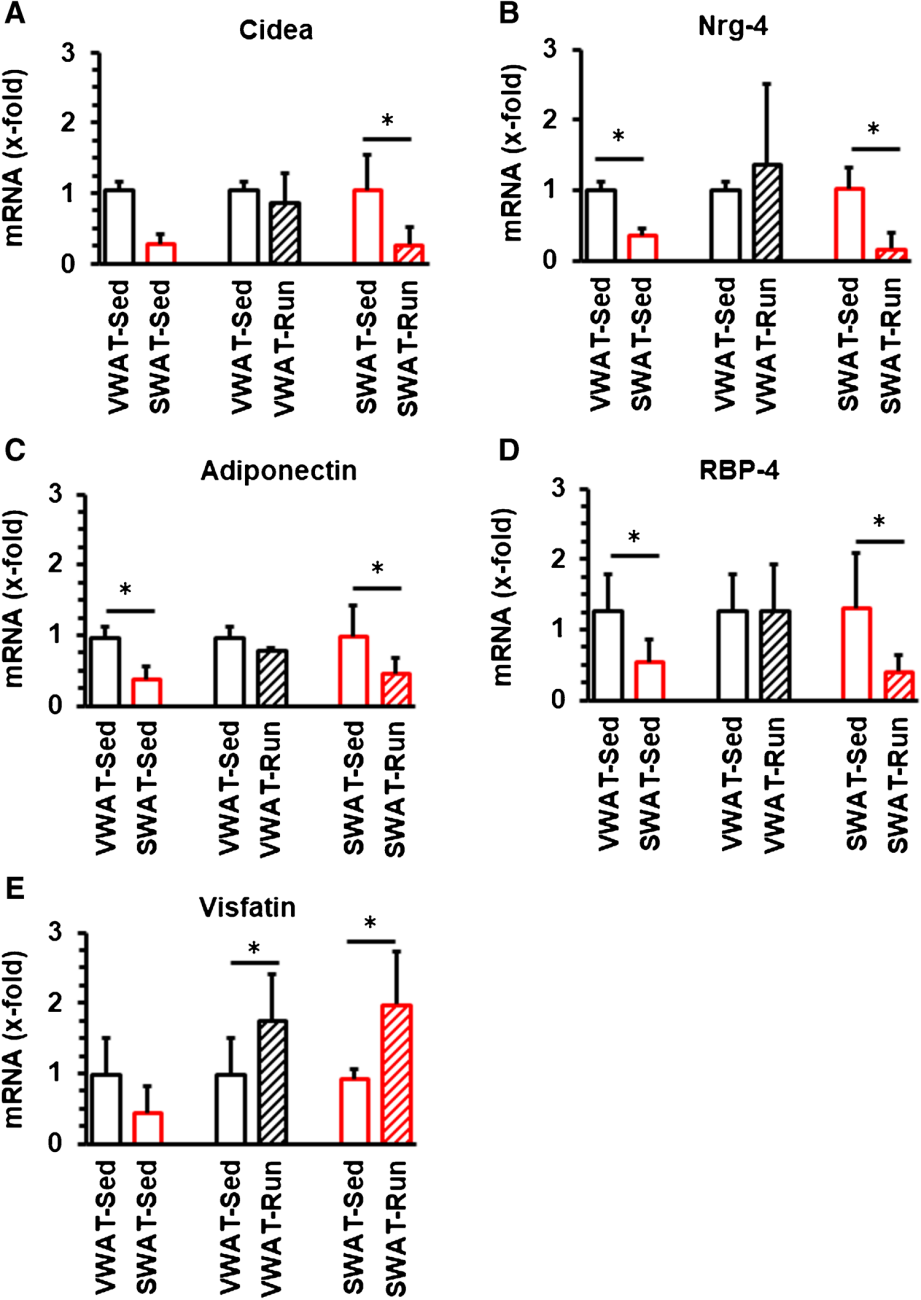
Expression of typical browning markers in VWAT and SWAT from normotensive Wistar rats and the effect of voluntary exercise (Run) compared to sedentary controls (Sed). Data are means + S.D.; *t*-tests were performed between two columns and indicated by *, if *p* < 0.05

**Fig. 4:**
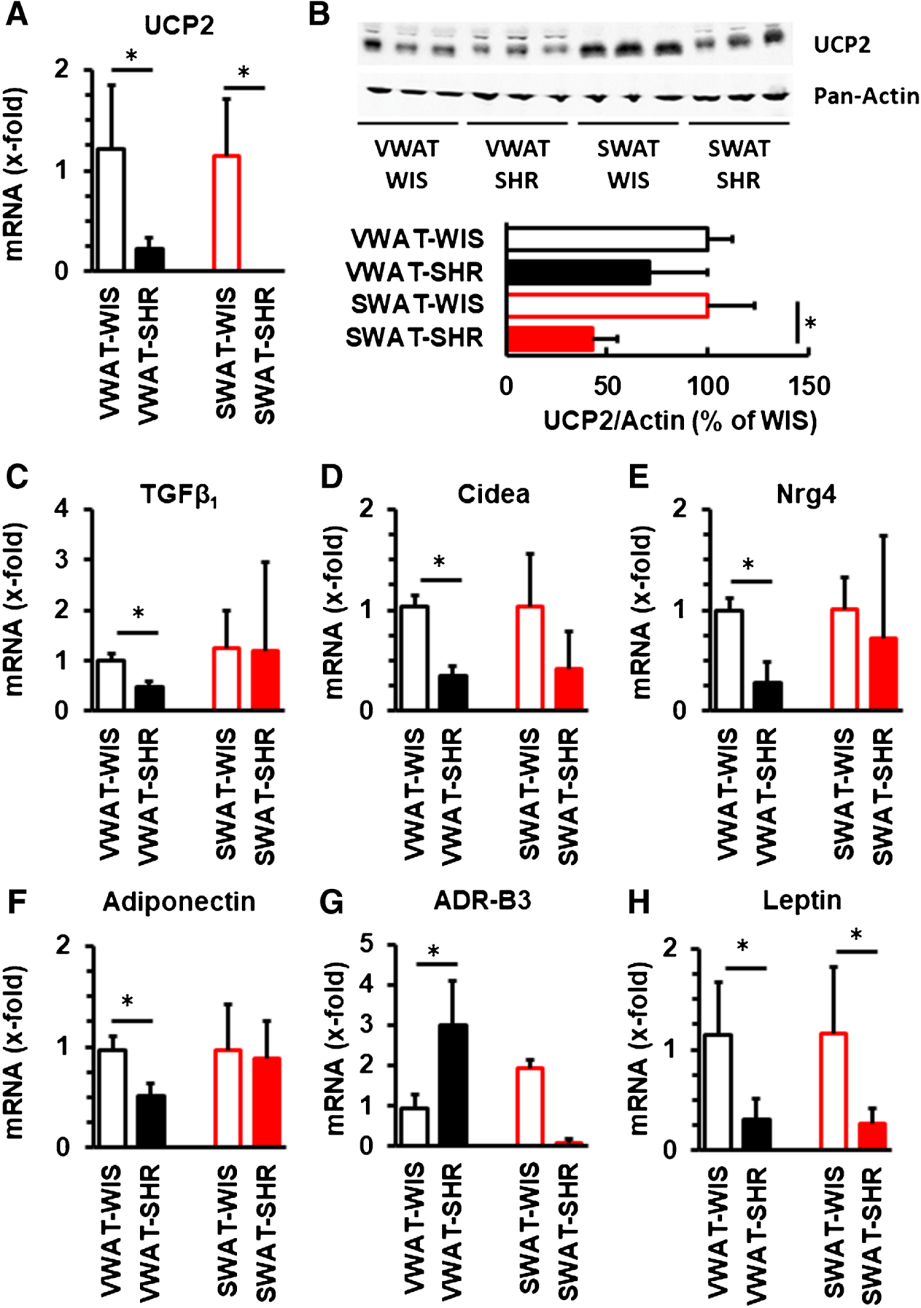
Expression of typical browning markers in VWAT and SWAT in normotensive rats (WIS) and spontaneously hypertensive rats (SHR). Data are given for mRNA (all) and protein (UCP2). Data are means + S.D.; *t*-tests were performed between two columns and indicated by *, if *p* < 0.05

**Fig. 5:**
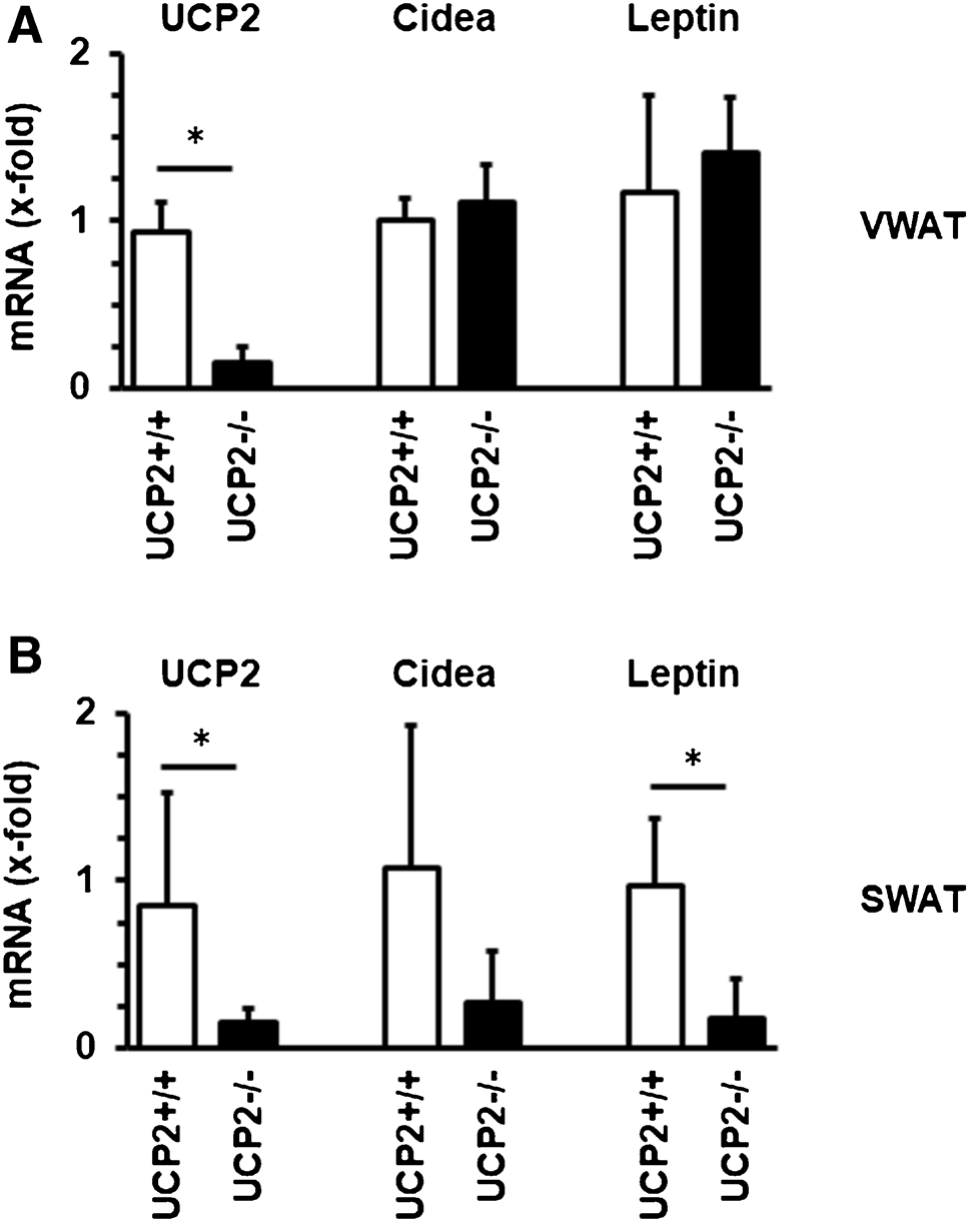
Effect of genetic depletion of UCP2 on the mRNA expression of Cidea and leptin in VWAT and SWAT. Data are means + S.D.; *t*-tests were performed between two columns and indicated by *, if *p* < 0.05

All data are presented as means ± S.D. *p* values were calculated by unpaired *t*-tests (Figs. 3 and 4) and *p* values below *p* ≤ 0.05 are indicated by asterisks. One-way ANOVA with Student-Newman-Keuls post hoc analysis was performed for data analysis of Figs. 2 and 5. Data pairs with *p* ≥ 0.05 are labeled with the same letter and data pairs with *p* ≤ 0.05 with different letters.

## Results

### Classical browning parameter in BAT and different types of WAT

First, we confirmed a high expression of UCP1 in interscapular BAT and also in WAT located in the immediate vicinity of BAT (Fig. 2). In contrast, UCP2, acting as a metabolic switch in myocardial cells [16], shows the opposite type of expression (Fig. 2). As expected, Cidea-1, known to act as an upstream activator of UCP1 expression [17], was strongly expressed BAT (Fig. 2). Neuregulin (Nrg)-4, an activator of erb-b2 receptor tyrosine kinase 4 [18], is also strongly expressed in BAT but not VWAT (Fig. 2). Collectively, these results demonstrate differences between WAT in close proximity to BAT and VWAT.

### Effect of exercise on the molecular signature of SWAT and VWAT in normotensive rats

The classical browning marker UCP1 could not be detected in either SWAT or VWAT. Therefore, we addressed the question whether the expression of browning markers distinct from UCP1 differs between SWAT and VWAT. The expression of several browning markers was analyzed in these two different types of adipose tissue in normotensive rats that were kept under standard housing conditions (sedentary conditions; VWAT-Sed and SWAT-Sed). Cidea-4 was present in three out of six samples in the VWAT but in all samples from SWAT. However, the level of expression of Cidea-4 in SWAT was lower than in Cidea-positive VWAT (Fig. 3). Similarly, expression of Nrg-4 (Fig. 3), adiponectin (Fig. 3), and retinol-binding protein (RBP)-4 was lower expressed in SWAT than in VWAT (Fig. 3). Visfatin, a member of the family of adiponectins, was detected in three out of six samples of VWAT and in all samples from SWAT but again at low level of expression in Visfatin positive samples from SWAT compared to VWAT (Fig. 3). Collectively, these data suggest that browning and metabolic stress markers are stronger expressed in VWAT than in SWAT.

Subsequently, we analyzed the effect of exercise on these markers. Voluntary exercise in normotensive Wistar rats affected the mRNA expression of Cidea, Nrg-4, adiponectin, and RBP-4 in SWAT but not VWAT (Fig. 3). All these four genes were downregulated in rats performing long-term exercise. In contrast, Visfatin, the fifth gene that was differentially affected by exercise, was commonly upregulated in SWAT and VWAT (Fig. 3).

### Comparison of the molecular signature of WAT between normotensive and spontaneously hypertensive rats

SHRs have occasionally been described as a model of metabolic dysfunction but whether this is accompanied by a different profile of mRNA expression in WAT remains unclear. Major differences between the expression of browning markers and metabolic molecules were found in WAT between both strains. The mRNA expression of UCP2 was lower in VWAT and SWAT from SHRs compared to normotensive Wistar rats (Fig. 4). In SWAT from SHR, UCP2 levels of mRNA were below the detection level and differences in protein expression in SWAT were stronger than in VWAT. As uncoupling proteins are of main interest in terms of energy consumption, we confirmed low expression of UCP2 on the protein level in VWAT and SWAT in tissues from SHR versus that of Wistar rats (Fig. 4). In addition, the expression of TGF-β_1_ (Fig. 4), Cidea (Fig. 4), Nrg-4 (Fig. 4), and adiponectin (Fig. 4) were all lower in VWAT from SHRs than in normotensive rats. The expression of adrenoceptor 3, barely detectable in SWAT from SHRs, was stronger expressed in VWAT of SHRs compared to VWAT from normotensive rats (Fig. 4). The expression of leptin was strongly reduced in VWAT and SWAT from SHRs compared to normotensive rats (Fig. 4). Collectively, these data suggest major differences in the molecular composition of VWAT between SHRs and normotensive Wistar rats and with the exception of leptin these differences were stronger in VWAT than in SWAT.

These data suggest that the strong decrease in UCP2 expression in SWAT from SHR, a protein located in the inner mitochondrial membrane, may trigger the different expression of Cidea and leptin in SWAT from SHRs. We tested this hypothesis in UCP2^−/−^ rats. The mRNA expression of UCP2^−/−^ was strongly reduced in VWAT and SWAT from knockout rats (Fig. 5). However, only in SWAT, but not in VWAT, this was accompanied by a similar decrease in Cidea and leptin expression as in SWAT from SHRs (Fig. 5).

### Effect of voluntary exercise on browning parameters in SHRs

**Fig. 6:**
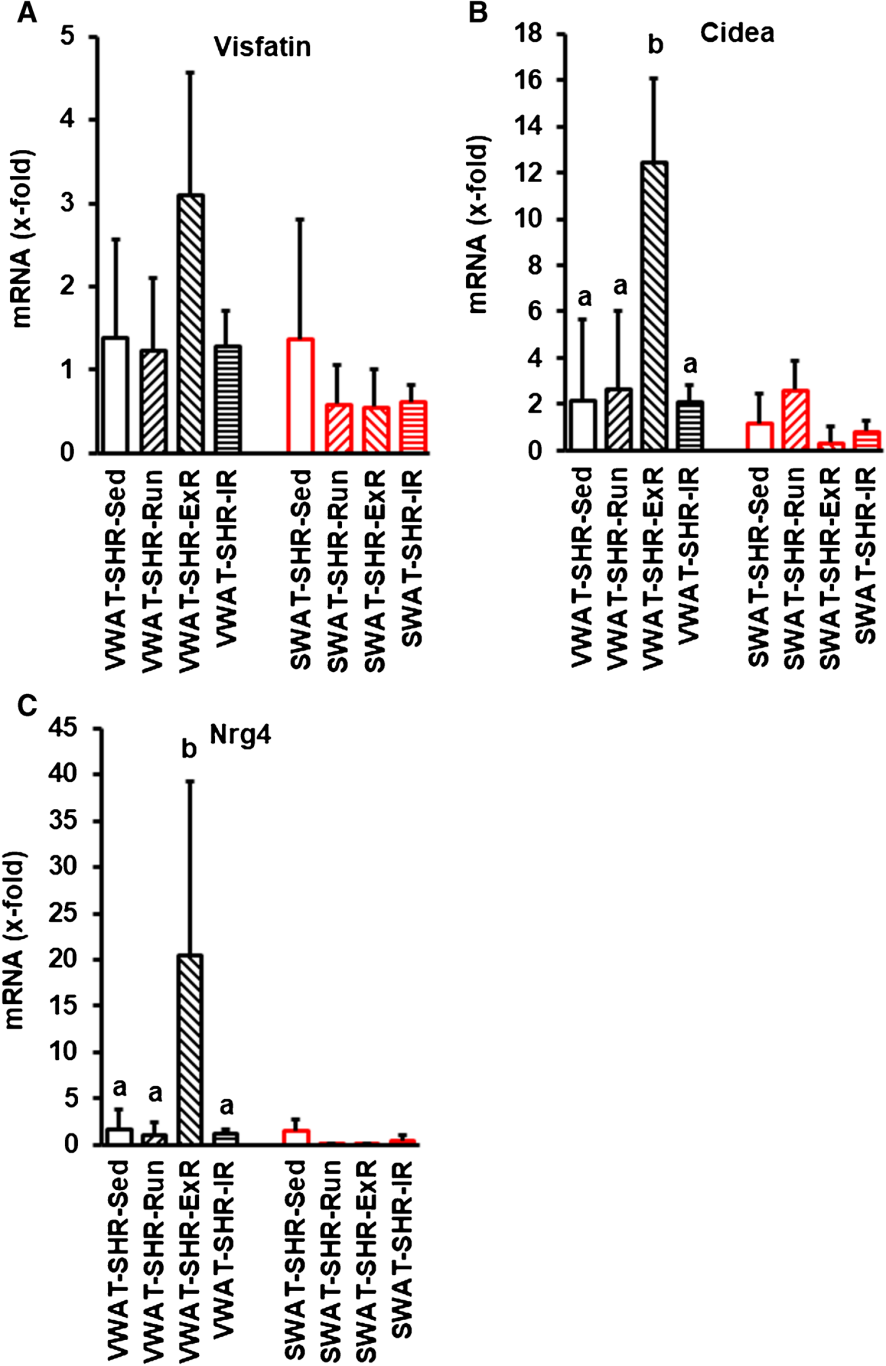
Expression of typical browning markers in VWAT and SWAT in spontaneously hypertensive rats (SHR) with different running protocols (Run, 10 months access to running wheels; ExR, 6 months access to running wheels and 4 months cessation; IR; free access to running wheels every second month). Data are means + S.D.; analysis was performed by ANOVA and Student-Newman-Keuls post hoc analysis. Groups are labeled with identical letters when the difference was >0.05. Different letters indicate group differences with *p* < 0.05

In normotensive rats, voluntary exercise significantly affected the expression of Visfatin, Cidea, and Nrg-5 in SWAT. However, in SHRs, SWAT did not respond to voluntary running activity in a similar way (Fig. 6). Unexpectedly, we observed an upregulation of Cidea and Nrg-4 and a similar trend for Visfatin in SHRs with cessation of free running wheel activity after 6 months (Fig. 6; ExR). No differences were obtained when rats had access to running wheels only every second month (interrupted running; Fig. 6; IR).

## Discussion

Our study compared SWAT and VWAT of normotensive and hypertensive rats and investigated the effect of exercise on these tissues in both strains. There are two main findings in our study. At first, recently discovered adaptations of SWAT in normotensive rodents to high physical activity do not allow to translate these findings to hypertensive rats. This result is important as many obese patients have hypertension as an important co-morbidity and our data suggest that metabolic improvements by high physical activity are more complex than expected from such studies. Second, our results show that a different responsiveness between SWAT and VWAT may be associated with reduced tissue expression of UCP2.

Regulation of energy metabolism of the whole body requires a fine-tuning between the main tissues involved in this process, namely, liver, skeletal muscle, and adipose tissue. Adipose tissue has several important physiological functions such as energy storage, energy homeostasis, thermoregulation, and regulation of appetite. Furthermore, it is involved in fine-tuning of the immune response and the cardiovascular system via the release of adipokines [19]. To cover all these functions, adipose tissues are widely spread throughout the body. However, different locations of adipose tissue go along with different phenotypes suggesting that different types of adipose tissue are linked to different functions. In terms of obesity, a dysregulation between energy intake and energy consumption leads to extension of adipose tissue size and changes in adipose tissue function. High physical activity should affect this dysregulation by improving energy expenditure. Adipose tissue is involved in the adaptation to high physical activity as it improves glucose metabolism and supports the energy requirements of the muscle by lipolysis [20–22]. Investigating the role of adipose tissue in response to physical activity, a lot of attention has been given to the mechanism of browning of WAT. The molecular marker of this process is UCP1 that is responsible for thermogenesis. We confirmed high expression of UCP1 and its upstream regulator Cidea in BAT. However, only WAT in the immediate proximity of BAT from the interscapular region had detectable expression of UCP1 whereas UCP1 could not be detected in either SWAT or VWAT. In some mice strains but not in mice in general, UCP1 expression as a measure of browning was induced by exercise. In this case, it was induced in UCP1 positive WAT [2]. This adipose tissue was termed “brite” adipose tissue indicating that brown adipocytes are present in WAT. High physical activity did not induce the expression of UCP1 in either SWAT or VWAT. In this aspect, our study conformed to previous reports that exercise does not induce browning of WAT in humans or rodents [20, 23–26]. Only few reports have reported conflicting results [3, 4]. From a mechanistic point of view, induction of UCP1 in adipose tissue in response to muscle work does not make sense as it further increases heat production. On the other hand, it has been argued that increased energy consumption is required to maintain proper fatty acid control under conditions of lipolysis. Some authors describe that re-browning of WAT is associated with less UCP1 expression in BAT [3, 4]. Furthermore, some results may be explained by heterogeneous behavior of different types of adipose tissues and does not exclude that other parts of the adipose tissue respond by re-browning. Whatever the explanation for these divergent results is, it is clear that a general re-browning of WAT by induction of UCP1 does not occur. In this aspect, our study is in line with other reports [20, 25, 26].

SWAT and VWAT differ not only in anatomic localization but more importantly in their physiological function. Specifically, the VWAT located in vicinity to organs like kidney, liver, and heart are of interest because they release adipokines that directly affect non-adipose tissues [1]. This was formerly studied for vascular adipose tissue and the impact of vascular adipose tissue on endothelial function. It is clear that different types of WAT require the expression of a different type of proteins to fulfill the different functions. In this study, we observed such differences for the expression of Nrg-4, adiponectin, and RBP-4 between SVAT and VWAT. Nrg-4 is known to be stronger expressed in VWAT than SWAT [27] as confirmed in this study. One of its physiological functions is to fine-tune the activity of adipose tissue and the liver [28, 29]. Adiponectin is an organ-protective protein that is exclusively expressed by adipocytes; however, its expression is markedly reduced in obese patients [19, 30]. RBP-4 is upregulated in obese rodents and contributes to insulin resistance. Collectively, the molecular expression of factors coupling adipose tissue with metabolism is lower in SWAT than VWAT suggesting that SWAT is acting more like an energy storage tissue and VWAT involved in adipose tissue-dependent control of metabolism. Exercise, as performed in this study by voluntary running wheel activity, strongly reduced the expression of such molecules in SWAT but not VWAT. In general, this conformed to earlier reports that SWAT is more sensitive to exercise than VWAT [1]. An exception to this rule is the upregulation of Visfatin in both types of adipose tissue. Mechanistically, induction of Visfatin in rats with high physical activity makes sense as it increases the glucose uptake in muscles [31]. The transcriptional regulation of Visfatin seems to be strain specific, as voluntary exercise increased Visfatin expression in SWAT and VWAT of normotensive rats but not in SHRs. This is a new finding from our study.

SHRs have high blood pressure and other cardiovascular risk factors such as insulin resistance [9]. In the current study, we first addressed the question whether SHR and normotensive Wistar rats differ in the expression on metabolism-related genes in WAT. In total, seven differentially regulated genes were identified, of which UCP2, adiponectin, TGF-β_1_, Cidea, Nrg-4, and leptin are lower expressed in VWAT from SHRs versus VWAT from normotensive rats. UCP2 expression in adipose tissue is depressed by vitamin D3 [32]. However, SHRs have lower vitamin D levels than normotensive rats but still lower UCP2 expression indicating other mechanisms leading to the low levels of UCP2 expression [33]. UCP2 is of specific interest as it may replace the classical browning marker UCP1 due to its structural similarity. Therefore, it is important that the lower mRNA expression was accompanied by lower protein expression as well. Importantly, selective knockdown of UCP2 in VWAT and SWAT caused similar changes in Cidea and leptin expression in SWAT but not VWAT. The data underline the importance of the protein of the inner mitochondrial membrane for the physiology of SWAT. The reduced expression of adiponectin and leptin in SHRs suggests that the impact of VWAT-derived hormones on energy homeostasis is less pronounced in these rodents. The lower expression of TGF-β_1_ in VWAT from SHRs, an anti-inflammatory cytokine of adipose tissues, suggests increased inflammatory stress. The lower expression of Cidea and Nrg-4 suggests an impaired regulation of glucose metabolism within adipocytes. However, the expression of adrenoceptor β_3_ was significantly increased. It is known that tyrosine hydroxylase activity in adipocytes from SHRs is more activate than in normotensive rats [8]. The upregulation of the corresponding receptors may simply reflect an over-activation of adrenergic stimulation in the adipose tissue. However, the norepinephrine-dependent lipolytic response of adipocytes from SHRs is lower than that of normotensive rats [34]. This may indicate that the observed differences are compensatory mechanisms to normalize lipolytic response to catecholamines. Due to the inability of catecholamines to increase lipolysis in adipocytes from SHR, exercise does not increase lipolysis in such cells [35] and we did not find an exercise-dependent increase in adrenoceptor-β_3_ expression. Most of the transcriptional differences between WAT from normotensive and hypertensive rats, except that of leptin, were specific for VWAT and barley seen in SWAT.

The aforementioned findings show significant differences in the molecular signature of adipose tissues in SHRs versus normotensive rats. Similar conclusions hold also for the effect of exercise on the molecular signature of SWAT and VWAT. More specifically, there was a lack of responsiveness with respect to Visfatin. While exercise did not affect the molecular signature in SWAT and VWAT in SHRs, cessation of exercise increased the expression of some browning makers in VWAT (Cidea, Nrg-4). One may assume that cessation of increased energy expenditure by exercise requires a proper compensatory upregulation of pathways that increase energy expenditure, but this requires further analysis to be properly confirmed. If these data can be translated into human behavior, it would suggest that high physical activity is more important for the regulation of adipose tissues than continuation of high physical activity.

Transcriptional adaptations of tissues to high physical activity are potentially triggered by the release of myokines from the skeletal muscle. However, such a release is typically seen after the onset of muscle work. In this case, we expected that alternation in physical activity as investigated by access to running wheels every second month (interrupted running, IR) would have a stronger impact on adaptation in fat tissue than permanent activity. That was, however, not the case. It might be that 4 weeks of running performance interrupted by 4 weeks of resting periods was too long to indicate such an effect as we have previously shown that an increased expression of IL-6 in skeletal muscles, an indicator of increased myokine release, was significant after 2 days but not in the IR group [13].

In conclusion, the current study conformed to two major findings from former research, namely, that exercise does not upregulate UCP1 in VWAT and SWAT and that exercise mainly affects the molecular signature of SWAT. A new finding of this study is that we identified an upregulation of Visfatin as a potential link between muscle load and adipose tissue function and show for the first time that this effect was abrogated in SHRs. Furthermore, the data analyzed in our two rat models show that SHRs have a phenotypic different VWAT and that exercise does not exert a similar adaptation in this strain compared to normotensive Wistar rats. The data may also explain the large discrepancy in the literature concerning the interaction between exercise and adipose tissue function. Genetic variability as between these two rat strains used in this study is remarkable and may affect the outcome.
